# Extracorporeal Life Support (ECLS) for Pulmonary Vein Stenosis Interventions

**DOI:** 10.1007/s00246-026-04434-6

**Published:** 2026-08-20

**Authors:** Stephanie M. Tsoi, Roberta L. Keller, Loren D. Sacks, Vinod Sebastian, Sanjeev A. Datar, Michael A. Smith, Jason Boehme, Jeffrey R. Fineman, Elena K. Amin

**Affiliations:** 1 Division of Critical Care Medicine, Department of Pediatrics, University of California San Francisco, San Francisco, CA, USA; 2 Division of Neonatology, Department of Pediatrics, University of California, San Francisco, San Francisco, CA, USA; 3 Division of Cardiology, Department of Pediatrics, University of California San Francisco, San Francisco, CA, USA; 4 Division of Pediatric Cardiothoracic Surgery, Department of Surgery, University of California San Francisco, San Francisco, CA, USA; 5 Cardiovascular Research Institute, University of California San Francisco, San Francisco, CA, USA

**Keywords:** Pediatric, Pulmonary vascular disease, Cardiac catheterization, Extracorporeal membrane oxygenation

## Abstract

Pediatric pulmonary vein stenosis (PVS) is associated with high morbidity and mortality. We describe a case series of pediatric patients with advanced multivessel PVS who underwent cardiac catheterization with veno-arterial extracorporeal life support (VA-ECLS) to prevent cardiac arrest around high-risk pulmonary vein angioplasty and/or stent placement. VA-ECLS provided hemodynamic and respiratory stabilization, allowing completion of technically complex interventions in critically-ill pediatric patients who may not have otherwise tolerated catheterization. Using VA-ECLS in this setting requires a thorough assessment of risks versus benefits and specific patient criteria for consideration. This series highlights the potential role of VA-ECLS as an adjunctive strategy during high-risk catheter-based interventions for pediatric PVS to improve quality of life and lengthen lifespan.

## Case Report

Mortality from pediatric pulmonary vein stenosis (PVS) is approximately 40% for 1–2 vessel disease and 80% for ≥ 3 vessel disease within 4 years of a diagnosis [1–3]. PVS has historically been considered inoperable as management with interventional procedures were temporizing and risky. Recently, management of pediatric PVS has advanced, and interventional cardiologists will now perform serial cardiac catheterizations for balloon angioplasty and/or stent placement. These high-risk procedures often require post-catheterization intensive care unit (ICU) admission and mechanical ventilation [4]. The catastrophic adverse event (AE) rate for patients with multivessel disease is approximately 12%; stroke is the most common major AE, while pulmonary hemorrhage and hemodynamic instability are amongst the potentially fatal AEs. AEs limit the scope of pulmonary vein interventions that can be performed during catheterization [5]. We describe our experience using veno-arterial extracorporeal life support (VA-ECLS) in children with severe PVS resulting in favorable outcomes, including our approach to risk assessment and VA-ECLS management.

*Patient 1* is an ex-30–4/7 weeks’ small for gestational age (SGA) male, with a ventricular septal defect (VSD) and patent ductus arteriosus (PDA), who was diagnosed with single vessel PVS at 1 month of age by echocardiography. A cardiac catheterization at 2 months of age revealed stenosis of the right upper pulmonary vein (RUPV), right middle pulmonary vein (RMPV), left upper pulmonary vein (LUPV), and left lower pulmonary vein (LLPV), with systemic right ventricular (RV) pressure. We performed multiple pulmonary vein (PV) balloon angioplasty with cutting and drug-eluting balloons. By 11 months of life, he had undergone five additional catheterizations (only one of which was complicated by pulmonary hemorrhage), as well as VSD and PDA device closure. He was then discharged home on non-invasive respiratory support, sirolimus, aspirin, bosentan and diuretics, returning for PV interventions every 3–6 months. At 23 months, he developed acute-on-chronic respiratory failure from pneumonia for which he required intubation. After antibiotic treatment, his RV pressure estimate had increased to systemic levels. Due to concern for hemodynamic instability during cardiac catheterization, his PV intervention was scheduled with ECLS backup. We performed stent angioplasty in the RLPV and LLPV with bare-metal stents while the surgery and perfusion teams were immediately available. Though the procedure was technically successful, he developed profound hypoxia and was ultimately cannulated onto VA-ECLS at the end of the procedure to prevent cardiac arrest. Being on VA-ECLS facilitated supportive interventions, therapeutic bronchoscopies, and lung recovery, enabling decannulation 3 days later. He is now 3 years old with no neurologic deficits and a pulmonary vascular resistance index (PVRi) of 2.9 iWU on ambrisentan, tadalafil, aspirin, sirolimus, supplemental oxygen, and non-invasive overnight continuous positive airway pressure (CPAP). RUPV, RLPV and LLPV are stented (drug-eluting/bare-metal) with angioplasty anticipated every 3–6 months (Fig. 1 A–H).

*Patient 2* is an ex-25–6/7 weeks’ SGA female with bronchopulmonary dysplasia diagnosed by cardiac catheterization with severe pulmonary hypertension (PH) with suprasystemic RV pressures without PVS at 3 months of age. At 5 months of age, imaging showed multivessel PVS that was confirmed on catheterization (PVRi 4–6 iWU, LUPV occlusion and severe stenosis of LLPV, RUPV, and RMPV), so cutting and drug-eluting balloon angioplasty was performed. After 3 more catheterizations, she was able to discharge home at 17 months of age on aspirin, sirolimus, sildenafil, subcutaneous treprostinil, diuretics, and non-invasive overnight CPAP. She returned every 3 months for catheterization with only one procedure complicated by pulmonary hemorrhage. At 22 months, she was admitted for respiratory failure concerning for PVS progression and an episode of prolonged hypoxia and hemodynamic instability prior to catheterization prompted VA-ECLS cannulation. We were able to perform focused interventions during cardiac catheterization while she was on VA-ECLS and successfully placed LLPV and RUPV stents. She was decannulated 6 days later without any adverse events or complications. Her follow-up brain magnetic resonance imaging (MRI) showed no evidence of hemorrhagic or ischemic injury. She is now 2.5 years old and continues on the same medication regimen as well as non-invasive overnight bilevel positive airway pressure (BiPAP). Her RLPV remains unaffected, while her RUPV and LLPV are stented (bare-metal). We anticipate that she will continue to need angioplasty every 3–6 months (Fig. 1 I–P).

*Patient 3* is an ex-37–5/7 weeks SGA male who presented with severe RV dysfunction at 10 months of age. An echo-cardiogram showed multivessel PVS and suprasystemic RV pressure. Given his hemodynamic instability from RV failure at presentation, he was electively placed on VA-ECLS before cardiac catheterization, which confirmed severe stenosis of all vessels. We successfully performed PV angioplasty, which included cutting and drug-eluting balloons. After the procedure, we initiated IV treprostinil, which was rapidly up-titrated given his hemodynamic support and stability on VA-ECLS. He was decannulated and extubated 2 days later. After 1.5 months, he discharged home on sirolimus, aspirin, bosentan, subcutaneous treprostinil, diuretics, and supplemental oxygen. The patient is now 2.5 years old, he has no neurologic deficits, has undergone 5 subsequent catheterizations, has a PVRi is 3.7 iWU, and all his PVs are patent. We anticipate that he will need repeat angioplasty every 6 months (Fig. 1 Q–X).

## Discussion

We present a case series of 3 children with multivessel PVS and severe PH who were supported with VA-ECLS around transcatheter PV interventions with good outcomes. All 3 of the patients received short-durations of VA-ECLS and had significant improvement in their venous obstruction and no adverse cerebrovascular events. These cases exemplify how VA-ECLS can facilitate successful interventions for pediatric patients with high-risk PVS, meaningfully extending the amount of quality life at home.

We developed prospective criteria for back-up availability or elective use of VA-ECLS prior to interventional PVS catheterization: (1) multivessel disease, (2) systemic or suprasystemic RV pressure, and (3) RV dysfunction. We also consider the overall likelihood of being able to comprehensively address the PV obstruction in order to optimize hemodynamics and improve quality of life. These criteria are similar to other institutions’ and are independent predictors of mortality from pediatric PVS [2, 6]. Together these criteria suggest that severe, multi-level stenosis contributes to long-standing obstruction of pulmonary venous blood flow and marked pulmonary vascular remodeling. Due to resultant increased pulmonary vascular resistance and decreased pulmonary reserve, these patients are at high-risk for developing RV failure and progressing to cardiac arrest during anesthesia induction and/or PV manipulation [7].

The potential risks and benefits must be considered carefully before deploying VA-ECLS. VA-ECLS requires systemic anticoagulation, which may increase the risk of pulmonary hemorrhage associated with interventions (e.g., mechanical vessel injury, rapid hemodynamic shifts following obstruction relief) [5]. However, the provision of VA-ECLS post-catheterization can allow stability during supportive interventions (bronchoscopy, high end-expiratory pressure). Stroke risk is elevated during both prolonged catheterization and VA-ECLS. We use bivalirudin on VA-ECLS (target PTT 60–80 s), and additional heparin during catheterization (target ACT ~ 300 s). In our cases, this approach prevented cerebral vascular events with limited sequelae from pulmonary hemorrhage. Other risks of VA-ECLS include prolonged sedation and vessel obstruction at the site of cannulation (although, our cases were all able to undergo successful right internal jugular and carotid reconstruction).

Optimal timing of cannulation requires an understanding of institutional resources. Case 1 exemplifies the importance of VA-ECLS standby, but had logistical challenges of cannulating at the time of hemodynamic instability in the middle of the cardiac catheterization. We used VA-ECLS in a more controlled setting pre-catheterization in Cases 2 and 3, and ultimately provided hemodynamic stability that decreased the total catheterization time. Review of stent placement for Case 1 versus 2 suggested that stable hemodynamics from VA-ECLS facilitated better stent placement.

We share our experience using VA-ECLS concurrently with interventional cardiac catheterization to manage high-risk PVS with favorable outcomes. This approach requires judicious and careful consideration of risks versus benefits of each individual patient by a multidisciplinary pediatric PVS and PH specialized care team.

## Figures and Tables

**Fig. 1:**
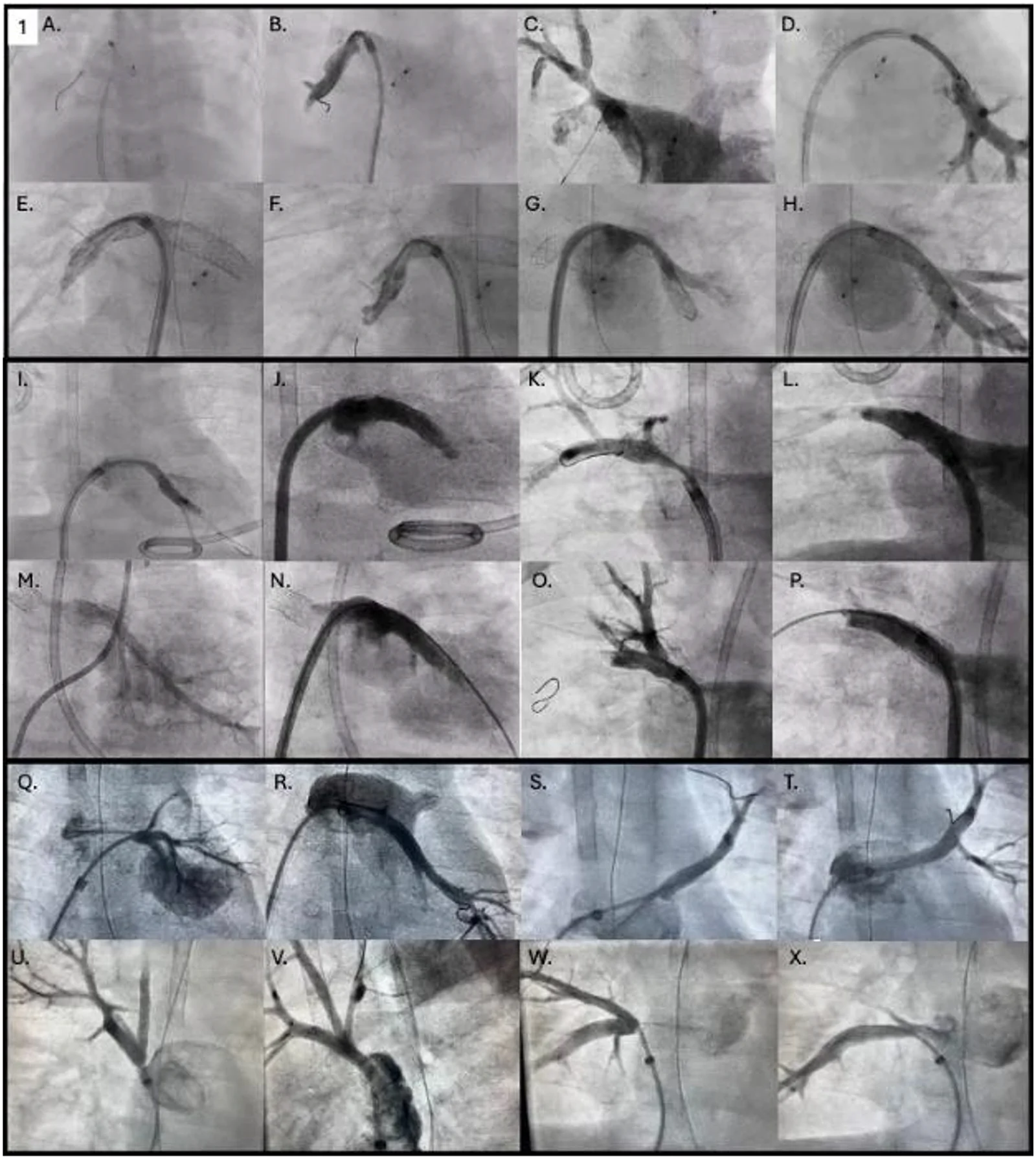
**A-D** Case 1: catheterization #10 at 23 months before going on VA-ECLS: **1A-1B**: RLPV stenosis, **1C**: RUPV stenosis, **1D**: LLPV stenosis with suboptimal stent placement. **1(E-H)** Case 1: catheterization #13 at 3 years old: **1E-1F**: RLPV with stent, **1G-1 H**: LLPV with stent in more optimal position. **1(I-L)** Case 2: catheterization #8 at 23 months while on VA-ECLS: **1I-1J**: appropriate stent positioning and length in LLPV and **1 K-1L**: RUPV, **1(M-P)** Case 2: catheterization #9 at 2.5 years old, improvement in LLPV and RUPV stenosis after cutting and drug-eluting balloon angioplasty, no additional stents required. **1(Q-X)** Case 3: catheterization #1 at 10 months while on VA-ECLS showing pre-intervention and post-intervention of cutting and drug-eluting balloon angioplasty, **1Q-1R**: LLPV, **1S-1T**: LUPV, **1U-1V**: RUPV, **1W-1X**: RLPV

## Data Availability

No datasets were generated or analysed during the current study.
